# Quantification of Extracellular–Polymeric Substances (EPS) Excreted by Foodborne Pathogen *Salmonella* Enteritidis PT30 During Biofilm Formation on Stainless Steel at Two Different Temperatures

**DOI:** 10.3390/microorganisms14061286

**Published:** 2026-06-06

**Authors:** Daniela Bermudez-Aguirre, Joseph Uknalis, Katrina L. Counihan

**Affiliations:** 1 Characterization and Interventions for Foodborne Pathogens Research Unit, United States Department of Agriculture, Agricultural Research Service, Eastern Regional Research Center, 600 East Mermaid Lane, Wyndmoor, PA 19038, USA; 2 Microbial and Chemical Food Safety Research Unit, United States Department of Agriculture, Agricultural Research Service, Eastern Regional Research Center, 600 East Mermaid Lane, Wyndmoor, PA 19038, USA

**Keywords:** cell attachment, food contact surface, sanitation, pathogens, proteins, carbohydrates, eDNA

## Abstract

*Salmonella* spp. is a pathogen of concern in the food industry. This study evaluated the effect of temperature (10 °C and 37 °C) on the biofilm formation of *S.* Enteritidis PT30 on stainless steel up to 50 h, assessing the biofilm index (*BI*). The main extracellular polymeric substances (EPS) such as carbohydrates, proteins and eDNA were quantified. Scanning electron microscopy (SEM) was used to evaluate the biofilm formation. Results showed the effect of temperature on biofilm formation and EPS production. After 5 h at 37 °C the growth of planktonic and biofilm cells considerably increased (~7 log); however, the *BI* was higher for biofilms at 10 °C. Carbohydrates reached a maximum concentration after 15 h at 37 °C (65.49 μg/mL). The protein concentration reached a peak after 24 h regardless of the temperature. eDNA production was similar between the two temperatures (α = 0.05) and through the incubation time, ranging from 1.65 to 2.21 ng/μL. SEM images showed three-dimensional structures of the biofilm formed at 37 °C composed of cells, filaments, excreted EPS and a network of fibers. These results can help to understand the biofilm formation from this pathogen and develop effective interventions to break down these complex structures and effectively sanitize food contact surfaces.

## 1. Introduction

*Salmonella enterica* serotype Enteritidis is a foodborne pathogen of concern worldwide. The presence of this microorganism has been widely reported in several food commodities generating outbreaks, illnesses and deaths [1,2]. Common food vehicles of *S.* Enteritidis are poultry meat, eggs and egg products, but it can be present in any animal food product [3,4,5,6]. The occurrence of this microorganism in food is not the only concern; it has been documented that the excellent ability of this specific *Salmonella* serotype to form strong biofilms in food processing environments creates cross-contamination problems [7,8,9]. The biofilms formed by this microorganism have been reported on a wide range of materials such as stainless steel, glass, polystyrene, polyethylene, polypropylene, granite, silicon, and nylon [2,7].

Biofilm formation is a complex and systematic process in which microbial cells produce compounds known as extracellular–polymeric substances (EPS) help to create the biofilm, allowing the cells to adhere to the surface, aggregate more cells, provide some protection against environmental stresses and, finally, form a biofilm [2,10,11]. The degree of EPS production is modulated by specific genes in the cell, and the specific serotype of *Salmonella* will determine these compounds. Some *Salmonella* serotypes are weak biofilm formers and can be easily eliminated with conventional sanitation processes. However, some serotypes, such as *S.* Enteritidis are known as strong biofilm formers likely because of specific genes that promote the production of EPS [7,12]. There are five main types of EPS, depending on their functions that are related to *Salmonella* biofilms: structural, sorptive, surface-active, active and informative [13]. EPS include polysaccharides, proteins or peptides, lipids, glycolipids, nucleic acids, and other compounds; the production of these is also affected by the incubation temperature and pH [1,8,13,14]. Some of the main components of EPS in *Salmonella* biofilms are curli and cellulose, while curli, a protein, promotes cell adhesion to the surface, cell aggregation and biofilm formation and cellulose has been associated with cell-to-cell interactions and resistance against environmental factors, offering a protective effect [2]. *S. enterica* handles environmental stresses by producing and releasing EPS [13]. However, the study of nucleic acids as part of EPS, such as extracellular DNA (eDNA), has not been fully explored yet.

Several interventions have been explored to sanitize and decontaminate food-contact surfaces in the industry. However, it has been observed that some of these interventions work well for only a specific microbial strain, allowing the possibility of other strains surviving in a rich food processing environment [7]. Several factors affect the removal of the biofilm, such as the incubation time and temperature, the presence and type of organic matter (i.e., food debris), the surface material, the serotype of the microorganism and the possible presence of background flora [10]. For example, *S.* Enteritidis is less resistant to chlorine treatment when the biofilm is formed at 4 °C, and it is explained in terms of cellulose production. When the cells are at low temperature, the production of cellulose is low and the protective effect to environmental agents such as chlorine is reduced [14]. Biofilm formation is not only a concern in food processing environments, but also inside storage chambers, refrigerators, crates, totes, shelves and any surface that can be in contact with food. Temperature can vary widely depending on the industry, facility, processing operations and geographic location. The quantification and identification of EPS under specific conditions of study can provide novel information that can be used to better understand the process of biofilm formation and develop effective sanitation interventions to break the biofilm and sanitize the food–contact surface.

To the best of our knowledge, the quantification of EPS from *S.* Enteritidis during biofilm formation has not been reported. Then, the aim of this research was to study the biofilm development of the foodborne pathogen *S.* Enteritidis on stainless steel at specific times and at two different temperatures (10 °C and 37 °C) identifying and quantifying some of the main EPS excreted by the cells. Scanning Electron Microscopy (SEM) was used to study the structure of the biofilm at specific incubation times and examine the mechanism of EPS excretion. A better understanding of the production and nature of EPS and the biofilm formation will allow for the correct search of natural sanitizers in the food industry.

## 2. Materials and Methods

### 2.1. Microbial Strain

A strain of *Salmonella enterica* serotype Enteritidis PT30 was used from the Culture Collection of the USDA ARS Eastern Regional Research Center (Wyndmoor, PA, USA). This *Salmonella* strain was chosen based on the strong biofilm properties exhibited in previous research [7]. A stock culture was kept at −80 °C, and to prepare the inoculum a thawed vial was used and a loopful (10 μL) of the microorganism was transferred to 5 mL of sterile Tryptic Soy Broth (TSB: MP Biomedicals, LLC, Solon, OH, USA) with 0.6% Yeast Extract (YE: Fisher bioreagents, Fair Lane, NJ, USA) (TSBYE) and incubated at 37 °C for 24 h. Cells were -re-cultured, centrifuged and washed to prepare the inoculum as previously reported [7]. The inoculum to study biofilm formation was adjusted to 10^3^ cfu/mL using TSBYE. Foodborne outbreaks have been reported because of the presence of biofilms with very low counts such as 10^3^ cfu/mL [15]. The cell viability of the inoculum was tested with Xylose Lysine Deoxycholate agar (XLD, Difco^TM^, Sparks, MD, USA). Plates were incubated at 37 °C for at least 48 h.

### 2.2. Biofilm Formation

Biofilm formation was studied on stainless steel (304 L) coupons to simulate a food–contact surface in industry. Coupons were purchased from Biosurface Technologies Corp. (Bozeman, MT, USA) and the dimensions are 1.27 cm (diameter) by 0.38 cm (thickness). Before using them, coupons were washed with tap water and alkaline liquid detergent Contrex (Decon Labs, Inc. King of Prussia, PA, USA). Coupons were sanitized with ethanol, rinsed and autoclaved as previously reported [7].

The biofilm formation was conducted inside polystyrene culture plates (12-well) purchased from Corning (Corning, NY, USA). Two ml of TSBYE with inoculum were added to each well, and one coupon per well was added with sterile tweezers. Two sets of plates were prepared at the same time. Plates were covered with lids and gently shake by hand for 5 s to allow good contact between the inoculum and the coupons. The culture plates were incubated at 37 °C and 10 °C inside storage chambers (MIR-154-PA, Panasonic Healthcare Co., Ltd., Tokyo, Japan) under static conditions.

### 2.3. Biofilm Quantification

To quantify the biofilm, a sample from each temperature was removed at specific times using sterile tweezers and transferred to 10 mL of saline solution (0.85% *w*/*v*) to remove planktonic cells. Then, the coupon was transferred to 5 mL of saline solution with beads and vortexed for 1 min to remove the biofilm cells. Both solutions containing planktonic and biofilm cells were serially diluted with BPW and plated on Aerobic Plate Count Petrifilm^TM^. All petrifilms were incubated at 37 °C for 24–48 h and counted using a Petrifilm^TM^ Plate Reader Advanced (Neogen, Lansing, MI, USA).

#### Biofilm Index

The biofilm index (*BI*) was calculated for specific points based on microbial growth. The *BI* was calculated as follows, as suggested by Nguyen et al. [16]:

(1) $$BI=\frac{Attached\;cells\;(\log cfu/{cm}^{2})}{Planktonic\;cells\;(\log cfu/{cm}^{2})}$$

using log-values (cfu/cm^2^) of attached and planktonic cells.

### 2.4. EPS Quantification

Three main components of EPS, were selected to be quantified in the biofilms formed by *S. Enteritidis*: carbohydrates, proteins and eDNA. Samples to quantify EPS were selected based on the biofilm growth data. Most of these points represented the regions in which an abrupt growth was observed (5, 15, 24, 40 h), as well as the beginning and the end of the biofilm formation (1 and 50 h). Coupons were immediately processed once they reached the specific time, removed from the storage chamber and were rinsed in phosphate-buffered saline (PBS) to eliminate any non-adhering bacteria. Then, coupons were placed in 1 mL of NaCl (1.5 M), with sterile glass beads and vortexed for 2 min. Afterwards, the liquid was filtered through a 0.22 μm membrane to remove bacteria and ensure a cell-free EPS solution. The filtrate was stored at −80 °C until used for EPS quantification.

#### 2.4.1. Carbohydrates

To quantify the carbohydrate portion of EPS, a glucose standard curve was prepared with D-(+)-glucose (Sigma, Saint Louis, MO) consisting of the following concentrations: 0, 10, 25, 50, 75, 100, 250, 500 and 1000 μg/mL. Twenty μl of each glucose standard was added into a 96-well plate and then 20 μL of each EPS extract was added. Afterwards, 20 μL of 5% phenol (Sigma, Saint Louis, MO, USA) was added to each well and then 100 μL of sulfuric acid (95%) (Sigma, Saint Louis, MO, USA). Each sample was processed in triplicate. The plate was shaken for 30 s in the plate reader to allow a homogeneous mix, and it was incubated for 2 h at room temperature (21 °C). The plate was read on a plate reader (Safire2, Tecan, Männedorf, Switzerland) at 492 nm. The carbohydrate concentration in the samples was calculated based on the glucose standard curve.

#### 2.4.2. Protein

Protein was quantified using bovine serum albumin (BSA) standards and Folin–Ciocalteu Reagent. The BSA standard curve included the following concentrations: 0, 1, 5, 25, 125, 250, 500, 750, 1000 and 1500 μL/mL. Briefly, 40 μL of each EPS sample and 40 μL of each BSA standard were added into a 96-well plate in triplicate. Then, 200 μL of modified Lowry reagent (Thermo Scientific, Waltham, MA, USA) was added simultaneously to each well. The microplate was mixed for 30 s, covered and incubated at room temperature for 10 min. Then 20 μL of Folin–Ciocalteu reagent (Thermo Scientific, Waltham, MA, USA) was added to each well and mixed for 30 s; the plate was covered and incubated for 30 min (room temperature). The absorbance was read at 750 nm in the plate reader. The protein content was calculated based on the albumin (BSA) standard curve and expressed as μg/mL.

#### 2.4.3. eDNA

Finally, the biofilm samples previously prepared for EPS were analyzed for nucleic acids. The extracellular DNA (eDNA) concentration in the EPS samples was measured with a DeNovix DS-11 FX+ spectrophotometer (DeNovix Inc., Wilmington, DE, USA) using the dsDNA application. The instrument was blanked with the extraction solution. A 2 µL aliquot of sample was placed on the sample surface and measured. Each sample was analyzed in triplicate, and the concentration was expressed in ng/μL of eDNA.

### 2.5. Scanning Electron Microscopy

Samples at specific times were chosen to be observed using Scanning Electron Microscopy (SEM) and again the samples were selected based on biofilm formation. For these samples, biofilms were prepared according to Section 2.2. After the specific time was reached, the liquid around the coupon was removed from the well. It was replaced with 2 mL of 2.5% (*v*/*v*) glutaraldehyde (Electron Microscopy Sciences, Hatfield, PA, USA) and left overnight to stop all cell activity. Samples were prepared for SEM as previously described by Bermudez-Aguirre et al. [10]. Briefly, samples were washed and dehydrated with serial solutions of ethanol and placed in a Critical Point Drying Apparatus (Denton Vacuum, Inc., Cherry Hill, NJ, USA), using liquid carbon dioxide (Welco Co., Allentown, PA, USA). Then, samples were mounted on stubs and sputter-coated with gold for 1 min (EMS 150R ES, EM Sciences, Hatfield, PA, USA). They were viewed with a Hitachi SU5000 Scanning Electron Microscope, (Hitachi High-Tech Corp., Tokyo, Japan) with an accelerating voltage of 3–5 kV.

### 2.6. Statistical Analysis

All experiments were conducted at least in triplicate on different days with a new and fresh inoculum. Microbial counts were based on at least three dilutions plated in duplicate. The basic statistical analysis (i.e., average, and standard deviation) was conducted using Microsoft Excel (Version 2501, Seattle, WA, USA). Furthermore, an analysis of variance (ANOVA—one way) was calculated using SAS (Version 9.4, Cary, NC, USA) with a confidence level of *α* 0.05 to determine any significant difference between microbial growth, EPS, and *BI*. Also, a pair-wise Tukey’s test was used to find significant differences between the tested conditions using *α* 0.05.

## 3. Results

The results of the present study will be discussed in three different sections one corresponding to the biofilm formation, one about the production of EPS and finally the sequence of images of the biofilm formation and EPS excretion according to time and incubation temperature.

### 3.1. Biofilm Formation

**Figure 1:**
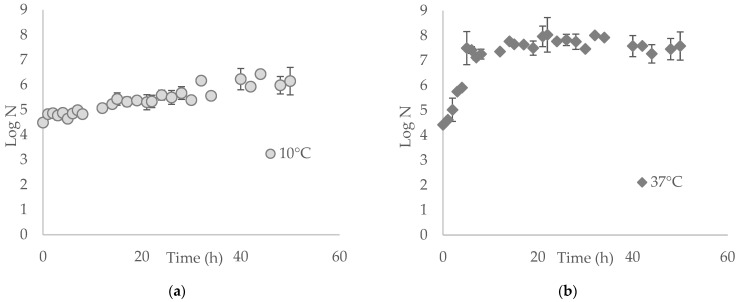
Planktonic cells of *S.* Enteritidis in stainless steel incubated at two different temperatures, (**a**) 10 °C and (**b**) 37 °C. Average values ± standard deviation.

**Figure 2:**
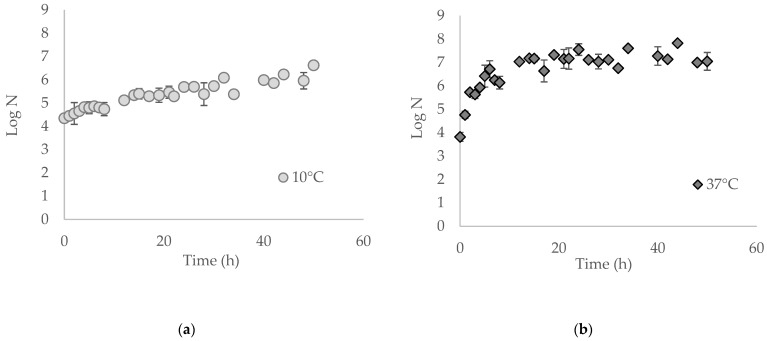
Biofilm associated cells of S. Enteritidis in stainless steel incubated at two different temperatures, (**a**) 10 °C and (**b**) 37 °C. Average values ± standard deviation.

The concentration of planktonic and attached cells during biofilm development is shown in Figure 1 and Figure 2, respectively. The influence of the temperature is clearly shown; the number of planktonic and attached cells at 10 °C increased at a slow rate, reaching a maximum growth (6 log) after 50 h (Figure 1a and Figure 2a). Meanwhile, the growth of planktonic cells and development of the biofilm at 37 °C was faster during the first hours of incubation (Figure 2b). There were specific times during which the cells showed growth increase. For the biofilm developed at 10 °C, there was no significant growth during the first hours (1 and 5 h) (Tukey’s test α = 0.05). The amount of *Salmonella* cells increased between 5 and 15 h and then did not significantly change through 24 h (Tukey’s test α = 0.05). Furthermore, the biofilm formation did not significantly increase between 24 and 40 h (Tukey’s test α = 0.05), but increased at 50 h. Regarding the biofilm formation at 37 °C, it shows a lag period during the first hour, with a significant increase by 5 h (6.4 ± 0.4 log), reaching maximum growth at 24 h (7.5 ± 0.2 log) and keeping similar levels by the end of the experiment (7.0 ± 0.3 log). Yang et al. [14] mentioned the favorable effect of temperature during biofilm formation and discussed the different scenarios, such as constant increase in biofilm density as the cells age, but also the stationary growth with a further decline. These authors mention that the composition, pH, and refreshment of the media might have an important role in the biofilm behavior. Gonzalez-Machado et al. [8] presented a study about the biofilm formation of *S.* Agona on polystyrene surfaces (37 °C) and the evaluation of the biovolume according to the time (up to 144 h). The largest biovolume of live cells was reported after 72 h with a further decline.

In a study published in 2012, fifty-one *Salmonella* Typhimurium strains were evaluated during biofilm formation and results showed that clinical, outbreak-associated and retail product cultures exhibited dense biofilms at 25 °C and in TSB at 35 °C, while industrial strains only showed dense biofilms at 25 °C and 1/20TSB [17]. The *Salmonella* strain used in the present research originates from a foodborne outbreak and presents a dense biofilm when incubated at 37 °C and TSB, which agrees with the mentioned study.

Biofilm formation is a process highly affected by the surrounding environment and tends to move to the equilibrium between planktonic and sessile cells. The biofilm index (*BI*) is a parameter to understand the dependence between the attached and planktonic cells. *BI* was calculated for the biofilm formed at 10 and 37 °C and the results are presented in Table 1. In general, higher values (close to 1.0) were observed at 10 °C. For the biofilm developed at a higher temperature, all except one value were below 1.0; however, the *BI* was the same (Tukey’s test α = 0.05) for the biofilms at 37 °C from 15 h to 50 h of incubation. These results agreed with previous studies conducted with the same *S.* Enteritidis PT30 strain in which the *BI* was always higher at 10 °C than 37 °C regardless of the material used for the food contact surface [7]. All *BI* were significantly different (*p* > 0.05) when compared at the same incubation time but different temperature, except for 40 h, which likely represents a mature biofilm in which the sessile (biofilm) cells at both temperatures did not depend on the planktonic cells surrounding the biofilm. During low temperatures, the biofilm formation is affected because of the changes in cell surface properties. There are limitations in the EPS biosynthesis and in the biofilm maturation. *Salmonella* reduces the slime matrix, depending more on cell-to-cell aggregation [18] (Vice 2025).

### 3.2. EPS Production

Available reports related to the EPS production are focused on several components such as polysaccharides or cellulose, lipids, and proteins. However, most of the reports dealing with quantification of EPS from *Salmonella* belong to research areas different from food processing environments such as medical applications. The results in this section will be presented in three main components, carbohydrates, proteins and eDNA production.

#### 3.2.1. Carbohydrates

The quantification of carbohydrates as EPS excreted by *S.* Enteritidis PT30 during the incubation time at 10 and 37 °C is shown in Table 2. The effect of temperature is again very clear in the higher and faster production of carbohydrates at 37 °C. The samples analyzed in the first two points, 1 h and 5 h, show very similar carbohydrate concentrations between both temperatures (from 23.53 to 32 μg/mL). However, at 15 h the excretion of carbohydrates at 37 °C doubled and reached 65.49 μg/mL. After 24 h the production of carbohydrates remained similar, and the concentration was equal (α = 0.05) according to Tukey’s test until the end of the incubation time indicating stabilization. Meanwhile, lower temperature (10 °C) samples kept constant carbohydrate production with a maximum peak at 24 h (33.84 μg/mL). Structural EPS are mainly composed of polysaccharides and proteinaceous components; some of these carbohydrates include cellulose, and colanic acid. Some cellulose-positive strains of *S.* Typhimurium produced fibrils (12–20 nm wide) that developed from all around the cell [13]. In a different study conducted with three strains of *S.* Enteritidis (ATCC 13076, 124 and 125) no cellulose was reported when the biofilm was allowed to grow at 4 °C. Authors concluded that temperature had an effect on cellulose production, but also the pH, with neutral and alkaline pH being more favorable to produce cellulose [14]. In the present research, there was some carbohydrate production at 10 °C, but it was very low compared to 37 °C, and the pH of the TSBYE was about 7.3, which is a neutral value. The composition of EPS excreted from *Salmonella* species is also influenced by the substratum on which the biofilm is formed, as Ledeboer and Jones [19] discussed. These researchers also presented the polysaccharide quantification of four *S.* Typhimurium strains that grew in Lennox agar plates, going from a very low concentration (1.4 μg/mL) to a peak concentration of 31.7 μg/mL when a mutant *Salmonella* strain (BJ3458) was used, using animal tissue. Gonzalez-Machado et al. [8] reported a low volume of β-polysaccharides when *S.* Agona was allowed to form a biofilm in plastic after 3 h, but the highest peak was reached after 48 h of accumulation at 37 °C.

#### 3.2.2. Proteins

Regarding proteins, the concentration was higher than carbohydrates in both temperature treatments. The concentration steadily increased with a peak at 24 h, showing concentrations of 68.41 μg/mL and 86.66 μg/mL for 10 °C and 37 °C, respectively, as shown in Table 3. After 24 h, the protein concentration gradually declined in both the 10 °C and 37 °C samples but remained higher than the carbohydrate concentration. Gonzalez-Machado et al. [8] found a strong positive correlation (*p* < 0.01) between the volume of the biofilm, live cells, and protein production when studying the biofilm formation from *S.* Agona at 37 °C. They suggested that the proteins provide the biofilm with structural integrity that requires intricate interactions between these molecules and carbohydrates, while the latter are more related to protective effects. As part of the structural EPS, curli fibers play an important role in biofilm production. It is a proteinaceous component, and these amyloids form rich pellicles allowing the cell to attach to surfaces such as Teflon and stainless steel, promotes surface colonization, but also cell-to-cell interactions [13,17,20]. The presence of amino acids during biofilm formation favors the production of curli and cellulose [20]. However, the strain is again an important factor in the production of curli. Several *S.* Enteritidis strains were evaluated under the same conditions and some produced curli and cellulose, others only curli, and other groups did not produce any of them [21]. Furthermore, some of the active EPS are enzymes excreted by microorganisms that regulate different processes during biofilm formation, such as degradation, synthesis or changes in molecules. However, there is still a lack of elucidation regarding this [13].

#### 3.2.3. eDNA

Nucleic acids are part of the EPS that have been recently included in several research reports, eDNA being the most studied acid in biofilm formation. In the present work, eDNA was quantified for *S.* Enteritidis PT30 during the biofilm formation at both temperatures. The concentration of eDNA is presented in Table 4, for the lower temperature (10 °C), there was an initial high production of eDNA, and the average values were statistically equal (α = 0.05) for the first hours of incubation (1, 5, 15 and 24 h), until a change was observed after 24 h with a decrease in the last hours of incubation (40 and 50 h). For the higher temperature (37 °C), the eDNA concentration was statistically equal (α = 0.05) during all the incubation times. The eDNA concentration went from 1.65 ng/μL to 2.21 ng/μL. Although eDNA quantification has not been reported for *Salmonella* species in food processing studies, to the best of our knowledge, other EPS that do not show important changes during biofilm formation are lipid production. The biofilm formed by *S.* Agona on a polystyrene surface presented very low production of lipids when compared to proteins and β-polysaccharides and remained almost constant from 3 to 144 h [8].

There are only few reports available about the functions of eDNA as part of EPS and more research needs to be conducted to understand the function of this component during biofilm formation. According to Maruzani et al. [13], eDNA forms part of the informative EPS. These authors discussed the identification of eDNA in biofilms (2 days-old) from a couple of *Salmonella* strains in which this component had a biofouling effect, although eDNA might also be linked to structural function. Özdemir et al. [12] discussed the functions of eDNA in biofilms such as providing nutrition and energy to the sessile cells, inducing horizontal gene transfer, helping in the general structure but also participating in the antimicrobial resistance. The same research team [12] presented the first evidence that eDNA might have an inhibitive or stimulative effect during biofilm formation, depending on the *Salmonella* strain and environmental conditions. From the seventeen *Salmonella* serovars tested in the study, authors reported the effect of eDNA as 62.9% of biofilm eradication for *S. Enteritidis* DMC3. However, they also mentioned the need to identify the specific factors for inhibition or stimulation studying the synthesis and release of eDNA during the biofilm process. The present research only quantified the eDNA during biofilm formation and further studies are required to clarify the role that this EPS component has during the full process.

### 3.3. Scanning Electron Microscopy

**Figure 3:**
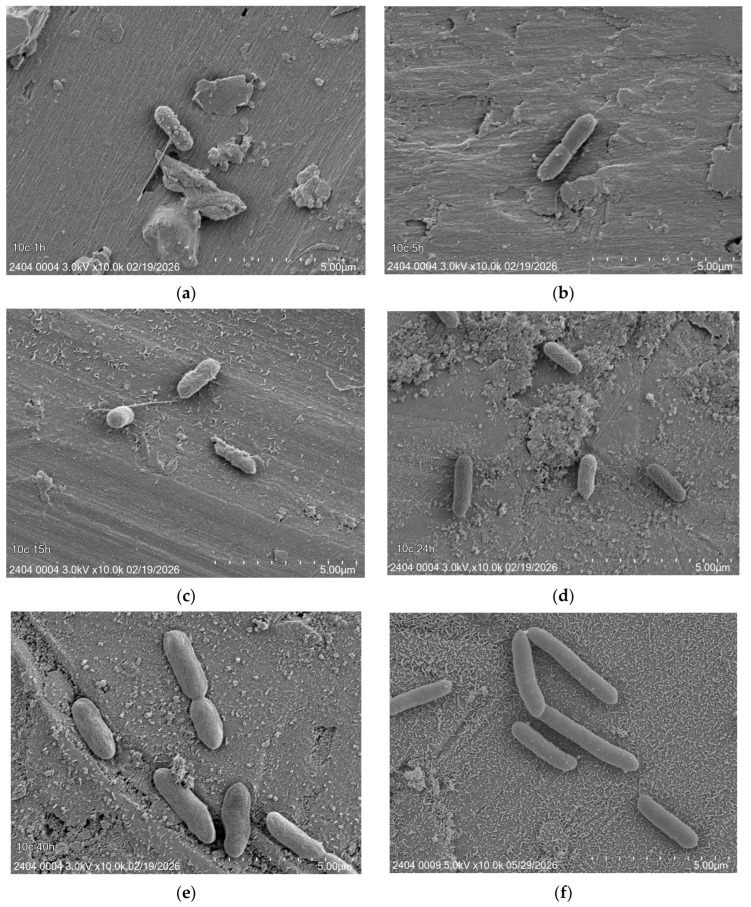
Images of *S.* Enteritidis biofilm attached to stainless steel coupons at different time intervals and incubated at 10 °C (Magnification 10K for all the images). (**a**) 1h, (**b**) 5 h, (**c**) 15 h, (**d**) 24 h, (**e**) 40 h, (**f**) 50 h.

**Figure 4:**
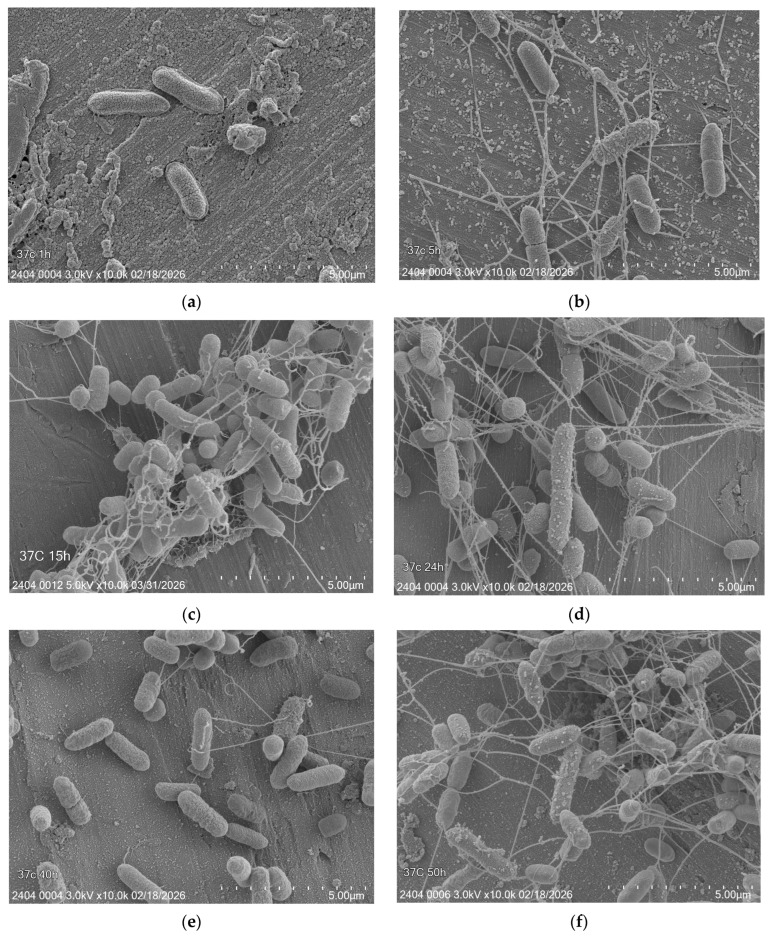
Images of *S.* Enteritidis biofilm attached to stainless steel coupons at different time intervals and incubated at 37 °C (Magnification 10K for all the images); (**a**) 1 h, (**b**) 5 h, (**c**) 15 h, (**d**) 24 h, (**e**) 40 h, and (**f**) 50 h.

**Figure 5:**
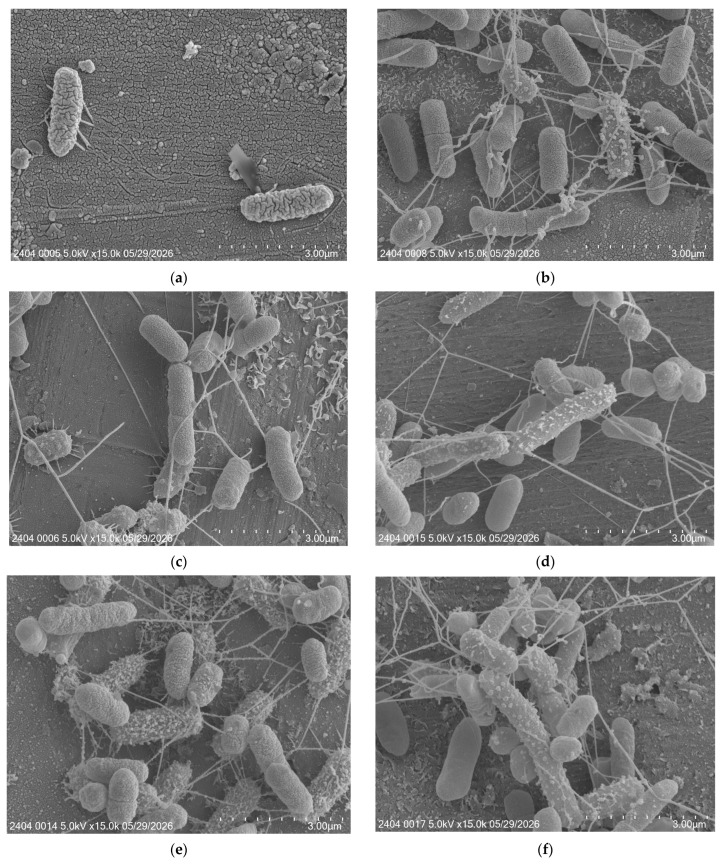
Electron microscopy images of *S.* Enteritidis biofilm cells in stainless steel incubated at 37 °C at different times showing the possible excretion of EPS (Magnification 15K for all the images). (**a**) 1 h, (**b**) 5 h, (**c**) 15 h, (**d**) 24 h, (**e**) 40 h, (**f**) 50 h.

The evolution of the biofilm was also monitored using electron microscopy and the images are presented in Figure 3, Figure 4 and Figure 5. Firstly, the images of the biofilm at 10 °C show very few cells on the surface of the stainless-steel coupons. Images are similar from times 1, 5 and 15 h (Figure 3a–c). More cells are observed by 24 h and some cell agglomerates started to be observed at 40 and 50 h (Figure 3e,f). Cells present similar morphology throughout biofilm formation, and they do not exhibit any filament or visible appendages attaching to the surface. Completely opposite behavior was observed at the higher temperature; the biofilms at 37 °C exhibited several changes according to the incubation time. In Figure 4b,c, the images observed at times 5 and 15 h already show the proliferation of several cells, but also the presence of some filaments connecting the cells. The image at 24 h presents a very well-connected network of cells (Figure 4d), attached by filaments, but it also shows the presence of small dots attached to the cell membrane. By the end of the incubation time (50 h), cell clusters are observed bonded by filaments, fibers, and more cells excreting EPS (Figure 4f). In the study presented by Gonzalez-Machado et al. [8], three main stages of biofilm formation were identified. The first phase (3–24 h) was identified by small clusters, micro-colonies and the formation of EPS matrix. The second phase (24–72 h) the biofilm presented a compact structure, and finally the third phase (72–144 h) showed an increase in dead cells.

Gong et al. [11] mentioned that EPS can be classified as bound or free; the first case corresponds to those compounds loosely around the cell surface and it is also known as capsular EPS. Meanwhile, free EPS are exuded from the cells and released into the surrounding environment; these are also known as soluble EPS. *Salmonella* EPS have been studied in other fields different from food processing and most of the data comes from research in medicine using different contact surfaces and conditions. However, it has been reported previously that the extracellular matrices produced by *Salmonella* are composed of curli and cellulose. Curli is composed mainly of amyloid fibers that allow the adhesion to the surface and cell aggregation. Meanwhile, cellulose promotes cell-to-cell interactions, adhesion to surfaces and environmental resistance [2]. It has also been documented that mature biofilms are more resistant to adverse physical and chemical conditions because of the three-dimensional structure composed for the biofilm cells embedded in EPS components [22].

The presence of small “dots” on the surface of the cells of *S.* Enteritidis is likely the production and excretion of EPS, mainly carbohydrates, that was boosted because of the effect of temperature. The images observed match very well the EPS production, according to testing time. In Figure 5, there are closer views (15K magnification) of cells belonging to the biofilm formed at 37 °C. The first image at time 1 h shows the *Salmonella* cells without visible changes on the cell membrane, although one of the cells shows some appendages attached to the surface (Figure 5a). However, for time 5 h the cell in the right of the image (Figure 5b) shows several of these “dots” on the surface and some of these structures seem to be the origin of the filaments that are bonding other cells but also attaching the biofilm on the surface. By 15 h, there is a considerable increase in these filaments forming a network not only of these new structures but also a complex cell system (Figure 5c). Based on the results of Table 2, this time is when the concentration of carbohydrates reached a maximum peak, so the presence of these filaments likely represents the excretion of these compounds. Figure 5d,e show more of these cells with dots in the surface and connecting the filaments. By the end of the incubation time, 50 h (Figure 5f), the image shows the presence of these “dots” along the filaments, representing another peak in the carbohydrates production, matching the results of Table 2 and showing some kind of saturation of carbohydrates. These networks of cells and filaments completely attached to the surface represent a food safety risk because the use of common sanitation methods might fail in the removal and full sanitation of food contact surfaces. Yang et al. [14] reported that the production of cellulose and curli depends also on the strain of *S.* Enteritidis, and the biofilm resistance to sanitizers is not fully understood, but it is believed that cellulose play a role in the resistance and might hinder the penetration of sanitizers. Shatila et al. [1] also mentioned the web-like network of *S.* Enteritidis TM6 attached to glass using fimbrial adhesins because of the production of cellulose, which according to these authors provides structure and maintains organization in the biofilm, while curli is more related to biofilm formation in the early stages. These complex systems of filaments and cells forming clumps might protect *Salmonella* from the effect of sanitizers. If these filaments are composed of cellulose and curli, sanitizers able to break down these molecules should be tested to disrupt these biofilms.

In an early study about biofilms formed by *S.* Enteritidis the presence of large quantities of intracellular polysaccharide were identified. Transmission electron microscopy showed the presence of cytoplasmic granules along the cytoplasm, representing stored glycogen accumulated during the pre-incubation stage. The presence of glucose in the medium also increased the formation of granules and was linked to higher virulence [23]. The microbial medium used to conduct the biofilm formation in the present study was TSB, according to the manufacturer it contains 2.5 g/L of dextrose [24], that could help in the carbohydrate production.

## 4. Conclusions

Biofilm formation and production of EPS from *S.* Enteritidis PT30 was greatly influenced by the temperature. A mature biofilm was observed after 24 h of incubation at 37 °C. The biofilm index showed higher values at the lowest temperature (10 °C). Carbohydrates, proteins and eDNA were quantified from the biofilms at 10 °C and 37 °C. Carbohydrates showed an important increase in concentration after the first hours of incubation at 37 °C, while protein concentration reached a maximum peak after 24 h regardless of the temperature. eDNA did not present important changes according to the time or temperature between the incubation conditions chosen for the present manuscript. The use of SEM helped to better understand the development of the biofilm but also exhibited the formation of complex networks between sessile cells, cellulose, curli and likely the excretion of macromolecules from the *Salmonella*.

Nevertheless, there are still several research needs to investigate such as the role of eDNA and elucidating its synthesis and release mechanism during biofilm formation and the identification and quantification of other important EPS from *S.* Enteritidis such as lipids. These results can help to develop better interventions to break down biofilms composed of *S.* Enteritidis and search for novel sanitizers that can act effectively in cell inactivation.

## Figures and Tables

**Table 1:** Biofilm Index (*BI*) for *S. Enteritidis* on stainless steel at two different temperatures.

Time (h)	*BI* (10 °C)	*BI* (37 °C)
1	0.92 ± 0.00 ^a^	1.03 ± 0.03
5	1.03 ± 0.02 ^ab^	0.86 ± 0.01
15	0.99 ± 0.00 ^ab^	0.94 ± 0.01 ^a^
24	1.02 ± 0.01 ^ab^	0.97 ± 0.02 ^a^
40	0.96 ± 0.06 ^abA^	0.96 ± 0.00 ^aA^
50	1.08 ± 0.08 ^b^	0.93 ± 0.02 ^a^

Average values ± standard deviation. ^ab^ Equal lowercase letters indicate statistically equal means (Tukey’s test α = 0.05) when compared between different times at the same temperature. ^A^ Equal uppercase letters indicate statistically equal means (Tukey’s test α = 0.05) when compared between two temperatures at the same time.

**Table 2:** Carbohydrate concentration (μg/mL) excreted by S. Enteritidis as extra-polymeric substance (EPS) during biofilm formation on stainless steel at two different temperatures.

	Carbohydrate Concentration (μg/mL)	Carbohydrate Concentration (μg/mL)
Time (h)	10 °C	37 °C
1	26.73 ± 1.85 ^ab^	23.59 ± 0.54 ^a^
5	23.53 ± 2.01 ^b^	32.00 ± 2.18 ^a^
15	22.70 ± 2.41 ^b^	65.49 ± 1.70 ^b^
24	33.84 ± 5.37 ^a^	54.29 ± 4.25 ^c^
40	23.04 ± 3.67 ^b^	53.96 ± 0.74 ^c^
50	33.63 ± 2.80 ^a^	59.08 ± 6.91 ^bc^

Average values ± standard deviation. ^abc^ Equal lowercase letters indicate statistically equal means (Tukey’s test α = 0.05) when compared between different times at the same temperature. All concentrations are significantly different (*p* ≤ 0.05) when compared between the two temperatures.

**Table 3:** Protein concentration (μg/mL) excreted by *S. Enteritidis* as extra-polymeric substance (EPS) during biofilm formation on stainless steel at two different temperatures.

	Protein Concentration (μg/mL)	Protein Concentration (μg/mL)
Time (h)	10 °C	37 °C
1	15.26 ± 0.47	44.89 ± 2.92
5	27.71 ± 1.58	52.15 ± 4.13
15	45.47 ± 0.74	74.13 ± 2.93 ^a^
24	68.41 ± 0.74	86.66 ± 0.55
40	51.94 ± 1.78	69.54 ± 2.62 ^a^
50	35.68 ± 1.16	59.66 ± 0.67

Average values ± standard deviation. ^a^ Equal lowercase letters indicate statistically equal means (Tukey’s test α = 0.05) when compared between different times at the same temperature. All concentrations are significantly different (*p* ≤ 0.05) when compared between the two temperatures.

**Table 4:** eDNA concentration (ng/μL) excreted by *S.* Enteritidis as extra-polymeric substance (EPS) during biofilm formation on stainless steel at two different temperatures.

	eDNA Concentration (ng/μL)	eDNA Concentration (ng/μL)
Time (h)	10 °C	37 °C
1	2.21 ± 0.22 ^bA^	1.88 ± 0.13 ^aA^
5	2.13 ± 0.15 ^bA^	1.89 ± 0.15 ^aA^
15	2.14 ± 0.13 ^bA^	1.99 ± 0.18 ^aA^
24	1.85 ± 0.12 ^abA^	1.69 ± 0.10 ^aA^
40	1.68 ± 0.17 ^aA^	1.65 ± 0.17 ^aA^
50	1.65 ± 0.12 ^aA^	2.02 ± 0.17 ^aA^

Average values ± standard deviation. ^ab^ Equal lowercase letters indicate statistically equal means (Tukey’s test α = 0.05) when compared between different times at the same temperature. Temperature. ^A^ Equal uppercase letters indicate statistically equal means (Tukey’s test α = 0.05) when compared between two temperatures at the same time.

## Data Availability

The original contributions presented in this study are included in the article. Further inquiries can be directed to the corresponding author.

## Abbreviations

The following abbreviations are used in this manuscript:

EPS	Extracellular-polymeric substances
BI	Biofilm Index
TSB	Tryptic soy broth
YE	Yeast extract
TSBYE	Tryptic soy broth with 0.6% yeast extract
XLD	Xylose lysine deoxycholate
CFU	Colony forming units
BPW	Buffered peptone water
BSA	Bovine serum albumin
PBS	Phosphate-buffered saline
SEM	Scanning electron microscope
ng	Nanogram
µl	Microliter
eDNA	Extra-cellular deoxyribonucleic acid
